# West Country Chest Society, Meeting in Exeter, March 1988

**Published:** 1989-02

**Authors:** 


### West Country Chest Society

### The Spring Meeting was held in the Post Graduate Centre at the Royal Devon and Exeter Hospital on Friday 4 March 1988

**The President Dr A R Tanser (Bath) took the chair.**

### RESECTION OF LUNG CANCER IN ELDERLY PATIENTS

The meeting opened with a paper by Mr J R Maneksha on behalf of Messrs Jeyasingham and Forrester-Wood. His paper consisted of a detailed analysis of the 'Peri-operative mortality and morbidity associated with total resection of lung cancer in elderly patients'. 50 consecutive patients aged between 70 and 85 were studied (43 males). There was great variability in pre-operative lung function, the majority having an $FEV_1$ between 40 to 80% predicted normal. Seven of the patients had pre-existing cardiac disease (stable controlled angina). The presence of superior mediastinal nodes detected on a CT scan did not necessarily constitute a contra indication for surgery and 17 of the 50 patients had dissection of the nodes with half requiring an intra pericardial dissection. Some patients also required partial excision and repair of the SVC, chest wall or diaphragm.

The post operative complications were reviewed in detail and interestingly only one patient required post operative ventilation. One patient died from myocardial infarction on the 14th post operative day and another from a cerabrovascular accident on the 6th day. The only other complications were a fatal oesophagopleural fistula and a severe pulmonary embolus from which the patient recovered. The 30 day mortality was 6% (3 out of 50) and Mr Maneksha recommended that surgery, even in this elderly age group, was justifiable so long as the patient was medically fit and the tumour was resectable.

### HYPOTHERMIC RESPONSE TO PARACETAMOL

Dr Eithne Mulloy (for Dr John Harvey) presented the case of a 68 year old man with a six week history of malaise and fever with night sweats. Chest x-ray was normal and there had been no response to Oxytetracycline or Amoxycillin. The only clinical clue that developed over the next seven days was of a very small right basal effusion, but a CT scan showed significant mediastinal lymphadenopathy, not apparent on the plain film. One of the striking features of this man was very high fevers with dramatic hypothermic reactions (T 32°C) following paracetamol. The fever was of the Pel-Ebstein type. Since the patient's general condition was deteriorating rapidly, a mediastinoscopy was performed and this revealed significant lymphadenopathy, histology of which showed mixed cellularity Hodgkin's disease. There was a very gratifying response to treatment with the MOPP regime (Prednisolone, Chlorambucil and Pro carbazine with intravenous Vincristine) with rapid weight gain and defervescence. None of the members had heard of this excessive response to paracetamol before.

### GASTRIC ASTHMA

Dr John Prior (Gloucester) presented a case of 'gastric asthma'. A 45 year old man who had had asthma since childhood, generally well controlled on Ventolin and Becotide Inhalers, gave a three year history of increasingly severe indigestion. Endoscopy had shown severe gastritis and peak flow monitoring had shown typical severe morning dipping in association with asthmatic exacerbations. There was no significant improvement with high dose inhaled or oral steroids or slow release Theophylline taken last thing at night. The patient was treated with an Angelchic prosthesis to control his reflux symptoms and also his nocturnal asthma. 24 hour intra oesophageal pH monitoring confirmed improved control of acid reflux. Dr Prior felt that 'gastric asthma' was an under diagnosed problem and said that he was going to study not only the effects of anti reflux measures on the control of asthma, but also the effects of asthma therapy on the control of reflux!

### BRONCHIAL ABSIDIA

Dr Susan Oxborrow (on behalf of Dr John Siddorn, Exeter) presented the case fo a 63 year old sheep farmer who presented with night sweats, malaise and purulent sputum with a chest x-ray showing quite marked bilateral consolidation. Eighteen months earlier he had suffered from an episode of bronchopneumonia, for which no cause had been found, but which seemed to coincide with the removal of a very dusty old lath/plaster ceiling. On this occasion the pneumonia appeared to respond to Benzyl Penicillin and Erythromycin, but because of persistent fever and night sweats a bronchoscopy was carried out which showed a clear bronchial tree exuding pus bilaterally. Persistent symptoms required the addition of Flucloxacillin and Gentamycin and repeat bronchoscopy simply showed an overgrowth with Candida. There was a steady deterioration in the patient's condition with no firm microbiological diagnosis. Speculative therapy was begun with broad spectrum antibiotics and Amphoteracin with Hydrocortisone cover. Subsequently, a MUCOR species of fungus was isolated from a sputum sample obtained prior to commencing therapy, which was later typed as Absidia Corymbifera.

Full investigation did not reveal any evidence of impaired immunity. The patient made a full recovery. Six months later he returned with an episode of haemoptysis, febrile symptoms and increased consolidation and Absidia was again cultured from his sputum. Subsequent episodes have been successfully treated with steroids alone and the theory is that this may, in part, be an immunologically based reaction to the presence of Absidia in the bronchial tree, although no precipitin antibodies had been detected to Absidia species or the patient's own isolate of the fungus. The patient remains well on a small dose of Prednisolone.

Dr Ivan Muscap (microbiologist) finally outlined the characteristics of MUCOR fungus species.

### MEDIASTINAL MASS

Mr Forrester-Wood (Frenchay, Bristol) presented a case of 'unusual mediastinal mass'. A 46 year old man presented with an irritating dry cough, noisy breathing and slight dyspnoea. Plain x-ray showed a large right sided superior mediastinal mass which was posterior and shown by CT scan and barium swallow to be compressing the trachea from the manubrium level to below the carina. Barium swallow had showed an irregular malignant looking indentation of the oesophagus, but biopsies of an haemorrhagic area in the oesophagus revealed only normal mucosa. A Tru-Cut biopsy of the lesion suggested a Schwannoma. Resection was considered a more appropriate treatment than radiotherapy and at thoracotomy a 14 cm tumour was found arising from the wall of the oesophagus. The tumour was resected and it proved possible to achieve primary closure of the resulting defect in the oesophagus. Subsequent special stains confirmed it to be a benign Schwannoma and not a leiomyoma. The post operative recovery was complicated at two weeks by a 2 mm oesophageal leak demonstrated on barium swallow, but this sealed itself following a further three weeks clinic feed nutrition and the patient was discharged well, taking a normal diet.

### CHRONIC PERICARDIAL EFFUSION

Dr Clive McGavin (Plymouth Health Authority) presented a case of 'chronic pericardial effusion'. A 53 year old woman with a chronic lymphocytic pericardial effusion, which had been present intermittently for at least 5 years but not causing cardiac tamponade, had received 9 months anti tuberculous chemotherapy for a positive tuberculin test. The chest x-ray showed shadowing compatible with pulmonary sarcoidosis, but Kveim test was negative. The cause of a chronic pericardial effusion and the management of this case were discussed. The likely diagnosis seemed to be chronic idiopathic pericardial effusion or sarcoidosis. A follow up report was promised.

### COLLAPSING STERNUM

Dr C M B Higgs (Royal United Hospital, Bath) presented a case of 'collapsing sternum'. A 74 year old female, with a 20 year history of productive cough, who had been on oral steroids for 2 years for rheumatoid arthritis, suffered a fractured pelvis in a fall. Over the next few months she complained of persistent central chest pain on exertion, unresponsive to treatment for oesophagitis and angina, but relieved by Temgesic. Compression fractures of T4 and T6 vertebrae with kyphosis were seen on x-ray. After admission for subacute bowel obstruction, due to constipation, she was noted to have breathlessness, green sputum, basal crackles and a tender sternum. An ununited sternal fracture was seen on new and previous x-rays. Despite treatment with local Lignocaine injections, there was rapidly progressive sternal buckling and severe kyphosis associated with breathlessness and foul sputum. Myeloma was excluded. $FEV_1$ 0.71, FVC 0.91. She was successfully treated by correction and stabilisation of kyphosis using long Luque rods fixed by wires to the dorsal vertebrae, T4-T10. She is now well at home, walking and pain-free. $FEV_1$ 0.51, FVC 0.71, but not breathless.

### NARCOLEPSY WITH APNOEA

Dr C M B Higgs (Royal United Hospital, Bath) presented the case of a 46 year old man with a diagnosis of narcolepsy, treated with Dexamphetamine. He presented with a lump of meat stuck in his oesophagus. Rigid oesophagoscopy was physically impossible and the obstruction was relieved by bouginage. Excessive somnolence and sleep apnoea were noted on the ward post-operatively. This had been a problem for 20 years. He had a bull neck, nasal speech and micrognathia. He had normal thyroid function, blood pressure, Hb, Pa 02 and lung function, although a smoker. Sleep studies identified recurrent prolonged falls in oxygen saturation, as low as 20%. He was essentially unable to breathe and sleep at the same time. CT scans and barium swallow screening showed complete obstruction of the naso-pharynx and pharynx during 'sleep'. An orthodontic appliance to fix the lower jaw in a forward position did not relieve symptoms. His symptoms of sleep disturbance and day-time somnolence, and the sleep apnoea have been completely abolished by the application of nasal CPAP using a pump and tightly fitting facial mask.

